# The prevalence of genetic variants of the Mas-related G-protein-coupled receptor X2 in patients does not explain perioperative hypersensitivity reactions to neuromuscular blocking agents

**DOI:** 10.3389/fphar.2025.1655722

**Published:** 2025-09-01

**Authors:** Alicja Dziadowiec, Mateusz Kwitniewski, Peter Kopac, Hubert Rybka, Lenka Sedlackova, Adriana Srotova, Wojciech Dyga, Ana Koren, Joanna Gluck, Radoslaw Kitel, Grzegorz Porebski

**Affiliations:** ^1^ Department of Clinical and Environmental Allergology, Jagiellonian University Medical College, Krakow, Poland; ^2^ Faculty of Biochemistry, Biophysics and Biotechnology, Jagiellonian University, Krakow, Poland; ^3^ University Clinic of Respiratory and Allergic Diseases, Golnik, Slovenia; ^4^ Medical Faculty, University of Ljubljana, Ljubljana, Slovenia; ^5^ Doctoral School of Exact and Natural Sciences, Jagiellonian University, Krakow, Poland; ^6^ Faculty of Chemistry, Jagiellonian University, Krakow, Poland; ^7^ GENNET, Prague, Czechia; ^8^ University Hospital Hradec Králové, Hradec Kralove, Czechia; ^9^ Center of Environmental Allergology, University Hospital in Krakow, Krakow, Poland; ^10^ Department of Internal Diseases, Allergology and Clinical Immunology, Medical University of Silesia in Katowice, Katowice, Poland

**Keywords:** genetic variants, mast cells, MRGPRX2, neuromuscular blocking agents, perioperative anaphylaxis, perioperative hypersensitivity

## Abstract

**Background:**

Neuromuscular blocking agents (NMBAs) may induce life-threatening perioperative hypersensitivity reactions (POH). In addition to the known IgE-dependent allergic background, a mechanism dependent on Mas-Related G Protein-Coupled Receptor-X2 (MRGPRX2) on mast cells has been postulated. This does not explain why NMBA-induced POH is sporadic and only occurs in some patients. We hypothesised that this phenomenon depends on single nucleotide polymorphisms (SNPs) in MRGPRX2 that enhance the response to NMBA.

**Methods:**

The protein coding sequence of the MRGPRX2 gene was sequenced in 31 patients with NMBA-induced POH and 42 controls. Medical history and skin tests were used to diagnose patients. Based on basophil activation test (BAT) and specific IgE, patients were classified as having a history of IgE-mediated or likely MRGPRX2-mediated reactions. Severity of POH was defined according to a commonly accepted scale. Molecular dynamics simulations were conducted to assess the functional and structural effects of the SNPs.

**Results:**

The most common causative drugs were rocuronium (n = 17) and atracurium (n = 7), the others were cisatracurium, vecuronium, suxamethonium (n = 2 each) and pipecuronium (n = 1). We detected one missense SNP: N62S, present in 38.7% of the study group and in 45.2% of the controls. Prevalence of this SNP in patients was not dependent on causative drug, BAT and sIgE results, or severity of POH or its skin manifestations. Analysis of root mean square deviation and fluctuation plots showed no significant differences between wild-type and N62S variants.

**Conclusion:**

SNPs detected within the protein coding sequence of the MRGPRX2 gene were not risk factor in NMBA-induced POH, regardless of the clinical characteristics of the patients and the causative drug in question.

## 1 Introduction

Perioperative anaphylaxis is a potentially life-threatening condition and one of the most common causes is neuromuscular blocking agents (NMBAs) (Garvey et al., 2019a). The known mechanism for these NMBA-induced reactions is mast cell degranulation mediated by drug-specific IgE (Garvey et al., 2019a; 2019b). However, NMBA-induced perioperative hypersensitivity reactions (POH) also occur in patients with no documented prior exposure to these drugs. Moreover in some of those who have experienced such reactions, an IgE-dependent background cannot be unequivocally confirmed (Ebo et al., 2023). Some light was shed on this issue by the study of McNeil et al., who showed that mast cell activation by NMBAs can occur independently of IgE via the MRGPRX2 receptor (McNeil et al., 2015). In their seminal paper, they showed that MRGPRX2 and its mouse ortholog Mrgprb2 were activated by rocuronium, atracurium, mivacurium, and a number of other compounds with structural similarities. The involvement of MRGPRX2 and Mrgprb2 in immediate drug hypersensitivity was demonstrated using knock-out animal models, as well as *in vitro* studies using human cell lines (McNeil et al., 2015). This initial observation was followed by other studies, which were frequently referenced in the current literature (Cao and Roth, 2023; Pichler, 2019; Porebski et al., 2018; Sabato et al., 2023).

However, these findings do not explain why NMBA-induced POH is sporadic and occurs only in some patients (Garvey et al., 2019a; 2019b). One of the proposed explanations is that single nucleotide polymorphisms (SNPs) in the MRGPRX2 gene alter the function of the receptor. Indeed, both loss-of-function (Reddy et al., 2017; Lansu et al., 2017; Chompunud Na Ayudhya et al., 2019) and gain-of-function SNPs have been described for MRGPRX2, the latter resulting in enhanced degranulation upon stimulation with the receptor ligand (Hamamura-Yasuno et al., 2022). Furthermore, Zhang et al. showed the disparities between populations in the frequency of perioperative anaphylaxis occurrence and suggested that genetic predispositions may be at the origin of these differences (Zhang et al., 2022). It is known that the way in which individuals respond to a given drug can be genetically determined (Kassogue and Fricke Galindo, 2024). To date, the search for a genetic basis has proved valid in explaining the causes of perioperative complications such as malignant hyperthermia or butyrylcholinesterase deficiency (Riazi et al., 2020) and, in the case of some severe drug-induced hypersensitivity reactions, has led to mandatory screening of certain patient groups for genetic risk factors for such reactions (Ferrell and McLeod, 2008). Although another research group recently published the results of MRGPRX2 genotyping in twelve patients who experienced hypersensitivity reactions to fluoroquinolones and vancomycin (Quan et al., 2025), no studies on NMBAs have been published to date.

## 2 Materials and methods

### 2.1 Study population

We studied a total of 31 adult patients with immediate perioperative hypersensitivity reactions to NMBAs, and 42 adult controls with a history of exposure to drugs of interest during general anaesthesia in the past without any hypersensitivity reactions. Clinical severity of POH was assessed using the modified Ring and Messmer grading scale (I-IV) classification as proposed by Garvey et al. (2019b). NMBA causality was confirmed in diagnostic work-up consisting of skin prick tests (SPT) and, if negative, intradermal tests (IDT) with drugs and other substances to which a patient had been exposed during perioperative period. The patients tested negative for other potential causes. Skin tests were performed according to routine methodology (Brockow et al., 2002). Maximal drug concentrations for skin tests were selected based on EAACI position paper (Garvey et al., 2019b). In addition to the positive skin test results, patients also underwent basophil activation test (BAT) and quantification of morphine-specific IgE (sIgE). All participants were of Caucasian race. Populations demographics and test results are summarized in Table 1. Based on the recently proposed algorithm (Sabato et al., 2023), patients with positive BAT and/or sIgE test were considered to have IgE-mediated reaction to NMBA, whereas patients with negative BAT and sIgE were considered to have likely MRGPRX2-dependent reaction. The study was approved by the Ethics Committees of Jagiellonian University (1072.6120.175.2020) and Golnik University (KME 150/09/13).

**TABLE 1:** Demographics and clinical characteristics of study subjects.

Control group n = 42	
Age (yr), mean (SD)	45 (15.5)
Female/male, n (%)	25 (59.5)/17 (40.5)
Patient group n = 31	
Age (yr), mean (SD)	52 (15.4)
Female/male, n (%)	20 (64.5)/11 (35.5)
Clinical manifestation, n (%)	
Skin/mucosal	26 (83.9)
Respiratory	13 (41.9)
Cardiovascular	27 (87.1)
modified Ringer – Messmer four step grading scale, n (%)	
Grade I	2 (6.45)
Grade II	7 (22.58)
Grade III	20 (64.52)
Grade IV	2 (6.45)
Serum tryptase level (ng/mL)	
Baseline median (range)	5.1 (2.9–8.0)
At time of reaction median (range) Relevant increase* n, (% of test performed)	43.4 (9.0–84.4) 8 (100)
Culprit drugs, n (%)	
Rocuronium	17 (56)
Atracurium	7 (23)
Cisatracurium	2 (6)
Vecuronium	2 (6)
Suxamethonium	2 (6)
Pipecuronium	1 (3)
Diagnostic tests against culprit drugs, n (%)	
SPT or IDT positive n (%)	31 (100)
BAT positive n (%)	11 (38)
sIgE morphine (kUA/L) median (range) positive, n (% of tests performed)	0.43 (0–4.75) 8 (26)

*>2 + 1.2 × baseline tryptase (Ebo et al., 2023), BAT, basophil activation test; F, female; M, male; IDT, intradermal test; SD, standard deviation; SPT, skin prick test; sIgE, morphine-specific IgE.

### 2.2 Laboratory assays

#### 2.2.1 DNA extraction and MRGPRX2 sequencing

Peripheral blood samples (2 mL) were collected from study participants in EDTA tubes. DNA was extracted from blood samples using the Genomic Midi AX Direct kit (A&A Biotechnology, Gdansk, Poland) according to the manufacturer’s instructions. The protein-coding region of the MRGPRX2 gene, which is located on a single exon, was amplified by polymerase chain reaction (PCR) and Platinum SuperFi II DNA Polymerase (Invitrogen). Primers were designed using Primer-BLAST tool (Ye et al., 2012) (NCBI Reference Sequence: NC_000011.10; forward: 5′-AAA​GTT​GCC​TCT​CAA​AGC​CAC​ATA-3′, reverse: 5′-ATG​AAA​ATC​AGT​GAA​CAT​GC AGC-3′). PCR products (1123 bp) were analysed on 1% agarose gels. Sanger sequencing was performed by Genomed (Warsaw, Poland). The SNPs were analysed in Mutation Surveyor software (SoftGenetics LLC, State College, PA), and followed by visual interpretation of all sequences.

#### 2.2.2 Basophil activation test (BAT)

Basophil activation was assessed by measuring CD63 antigen expression using the Flow2 CAST test (Bühlmann Laboratories AG, Schönenbuch, Switzerland). Blood samples were collected in EDTA tubes and processed according to the manufacturer’s protocol. Antibodies to the high-affinity IgE receptor and fMLP oligopeptides were used as a positive controls, while an unstimulated sample served as a negative control. Cells were stimulated with drugs solutions according to the manufacturer’s recommendations. A mixture of FITC-conjugated PE-conjugated anti-CCR3 antibody and anti-CD63 monoclonal antibody was added to the cell suspension, and were incubated at 37°C for 15 min, followed by addition of 2 mL of erythrocyte lysing solution was added. Samples were centrifuged, resuspended in 0.3 mL of wash buffer, and analysed within 1 h on a FACS Canto II flow cytometer (BD, Biosciences) with basophils gated as CCR3 +/SSC low cells. For patients recruited at the Golnik Clinic, heparinised blood was used for the test. Basophils were gated as CD123-positive and HLA-DR-negative cells, and the previously described protocol was followed (Kopač et al., 2021). Results were considered positive, if the percentage of CD63 positive basophils was ≥5% and the Stimulation Index (SI) was ≥2. SI was defined as the ratio of drug-specific basophil activation to background activation. Non-responders were excluded from analysis.

#### 2.2.3 Specific IgE measurement

Sensitisation to NMBAs is primarily assessed serologically by quantifying IgE reactivity to tertiary and quaternary substituted ammonium structures (Ebo et al., 2023). To quantify IgE specific for a quaternary ammonium compound, an IgE detection assay based on morphine sharing dominant epitopes with NMBA is available and frequently used (Dejoux et al., 2023; Takazawa et al., 2019). Specific IgE to morphine was quantified using the ImmunoCAP system (Thermo Fisher) according to the manufacturer’s instructions. The cut-off value was set at 0.35 kUA/L.

#### 2.2.4 Quantification of tryptase protein

As previously described elsewhere (Šelb et al., 2021), a commercially available ImmunoCAP assay with a lower detection limit of 1 ng/mL was used to measure tryptase levels in patients’ serum. The time of sampling for measurements was followed according to the recommendations of the European Academy of Allergy and Clinical Immunology, i.e., within 1–3 h after the drug reaction and more than 24 h for the determination of baseline tryptase levels (Garvey et al., 2019b).

### 2.3 Molecular dynamics simulation

Molecular dynamics (MD) experiments were performed using GROMACS 2024 (Abraham et al., 2015). The initial protein structure 7S8O was sourced from PDB database (Cao et al., 2021a; 2021b) and its ligand-binding, transmembrane domain (chain A) was used in the simulations. Only the non-terminal missing residues were repaired. N- and C-termini were capped with ACE and NME groups, respectively. Two separate systems were prepared–the first was a wild-type chain A, and the second was chain A with S62 introduced manually. These two structures were inserted into pure POPC bilayers and solvated with 0.15 M KCl in a rectangular bounding box. All of the system preparation was done using CHARMM-GUI (Jo et al., 2008; Lee et al., 2016; Wu et al., 2014), an online tool for generating MD input files. The force field applied was CHARMM36m. Prior to simulation, both systems were equilibrated in NVT ensemble for a total of 0.25 ns and then in NPT ensemble for a total of 1.75 ns. The production run lasted 500 ns and was carried out in NPT ensemble. The results of MD simulations were analysed using MDAnalysis Python library (Gowers et al., 2016) and visualised with PyMol (https://www.pymol.org/) (Schrödinger, LLC, 2025).

### 2.4 Statistical analysis

Results were expressed as absolute numbers and percentages for frequencies, and mean with standard deviations (if normally distributed) or median and range (if not normally distributed). Normality of the data was assessed using the Shapiro-Wilk test. A Student’s t-test was used to compare means. Pearson χ2 test with Yeats correction, when appropriate, or Fisher exact test was used to assess the differences in SNP frequencies between the patient and the control groups. P values less than 0.05 were considered statistically significant. All statistical analyses were performed using IBM SPSS Statistics 29 (PS IMAGO PRO).

## 3 Results

There were no statistically significant differences between the control and study groups with respect to sex (p = 0.665) and age (p = 0.09). In the patient group, skin/mucosal manifestations of POH (urticaria, itch, angioedema) were the most common, followed by respiratory and cardiovascular manifestations, with a clear predominance of grade III and IV reactions, as shown in Table 1. The most common drug responsible for POH was rocuronium, followed by atracurium. Two patients each had POH caused by cisatracurium, vecuronium, and suxamethonium, and one patient had a reaction caused by pipecuronium (Table 1). All patients tested had positive skin tests with the causative drug. Six patients had also positive results for both BAT and specific IgE. A further five had positive results for BAT only, and two had positive results for specific IgE only. BAT was not performed on two patients.

Analysis of the MRGPRX2 gene sequences of the patients and controls revealed a missense SNP: N62S (rs10833049) located in the first intracellular domain of MRGPRX2. Most SNPs were heterozygous, with only one patient having a homozygous SNP. There were no statistically significant differences in the percentage of N62S variant between the control group and the patients (Table 2). Comparison of the control group with selected subgroups of patients: (i) with negative BAT and sIgE, (ii) with mild to moderate symptoms (severity of reaction grade I-II), (iii) with hypersensitivity to rocuronium or (iv) to atracurium showed no significant differences in the proportions of N62S SNPs (Table 2). This SNP was also found in one patient after cisatracurium-induced POH and in one patient after vecuronium-induced POH.

**TABLE 2:** Comparison of the control group to the study subjects, in total and in the selected subgroups in respect of carriers of N62S genetic variant.

Control group, n (%)	Patients group, n (%)	p value
19 out of 42 (45.2)	Total 12 out of 31 (38.7)	0.945
	Patients with BAT and sIgE negative: 7 out of 18 (38.9)	0.649
	Patients with mild-to-moderate symptoms: 3 out of 9 (33.3)	0.714
	Patients with only cutaneous manifestation: 1 out of 2 (50.0)	1.000
	Patients with reaction to rocuronium 6 out of 17 (35.3)	0.774
	Patients with reaction to atracurium 4 out of 7 (57.1)	0.692

BAT, basophil activation test; sIgE, morphine-specific IgE.

We also observed no differences in the prevalence of N62S between (i) patients with IgE-dependent reactions to NMBAs and those with likely MRGPRX2-dependent reactions, (ii) patients with mild/moderate reactions and those with severe reactions (grade I-II and grade III-IV severity, respectively), and (iii) patients with only cutaneous manifestations of hypersensitivity and those with other clinical manifestations (with or without cutaneous symptoms and signs) (Table 3).

**TABLE 3:** Comparison of the patients selected subgroups in respect of carriers of N62S genetic variant.

Comparison between patients subgroups		p value
BAT and sIgE negative 7/18 (38.9)	BAT and/or sIgE positive 5/13 (38.5)	0.981
Mild/moderate symptoms 3/9 (33.3)	Severe symptoms 9/22 (40.9)	1.000
Only cutaneous manifestations 1/2 (50.0)	Other clinical manifestations 11/29 (37.9)	1.000

BAT, basophil activation test; sIgE, morphine-specific IgE.

**FIGURE 1:**
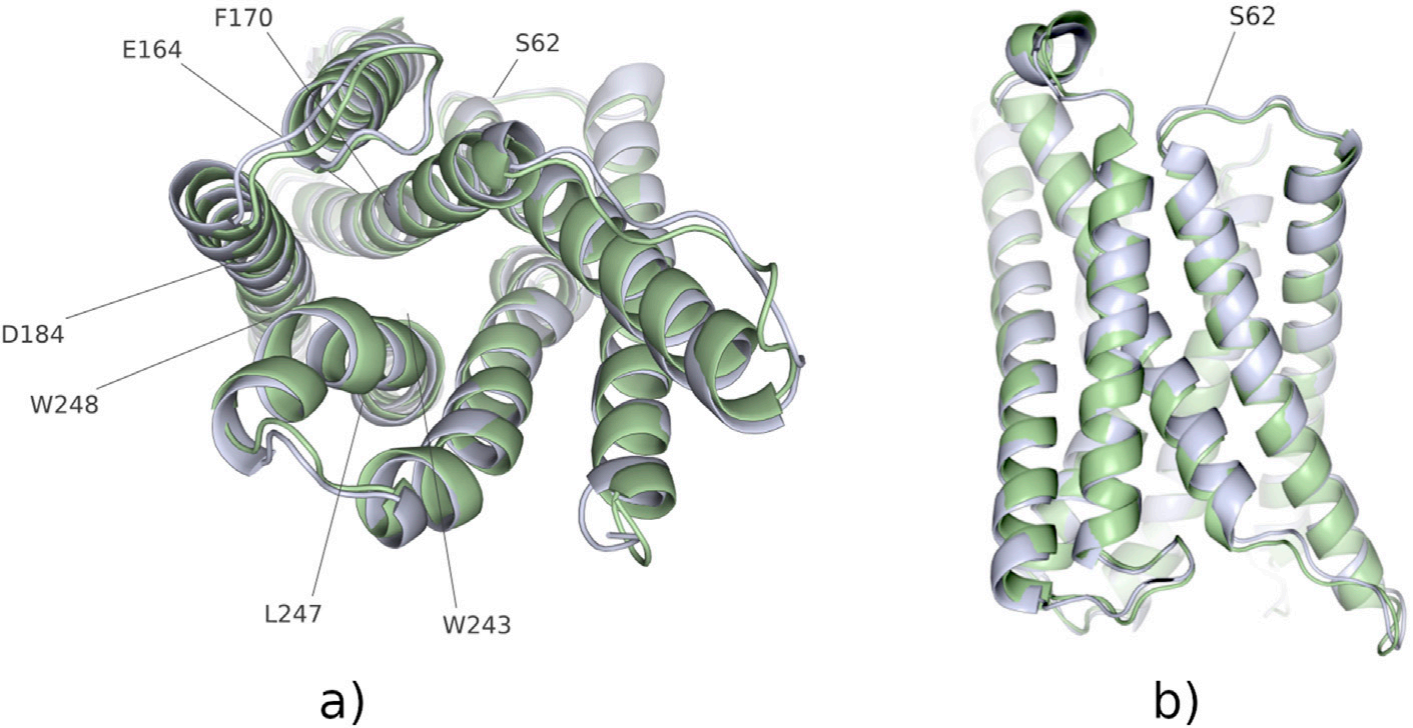
Superimposed 3D structures of the wild-type (blue) and N62S mutant (green) MRGPRX2 transmembrane domains. The structures were obtained by averaging atom positions over the entire MD trajectory and aligned in order to expose structural differences. **(a)** View on the binding site with subpocket 1 residues explicitly marked. **(b)** Direct view on the N62S mutation site (ICL1).

**FIGURE 2:**
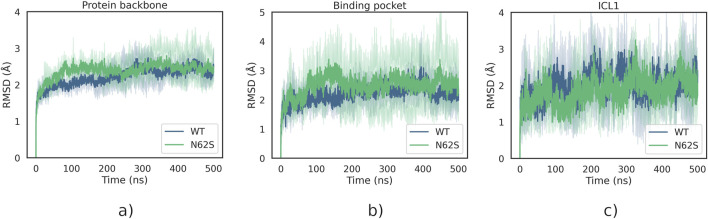
**(a)** RMSD plot of the protein backbone. **(b)** RMSD plot of the binding site, based on the subpocket 1 residues as identified by Cao et al. (2021a)\. **(c)** RMSD of the ICL1 region, in which the N62S substitution occurs. The RMSD values were calculated on α-carbon atoms and plotted as an average of three individual MD runs. ICL1; intracellular loop 1; N62S, variant Asn62Ser of MRGPRX2; RMSD, Root mean square distance; WT, wild type of MRGPRX2.

**FIGURE 3:**
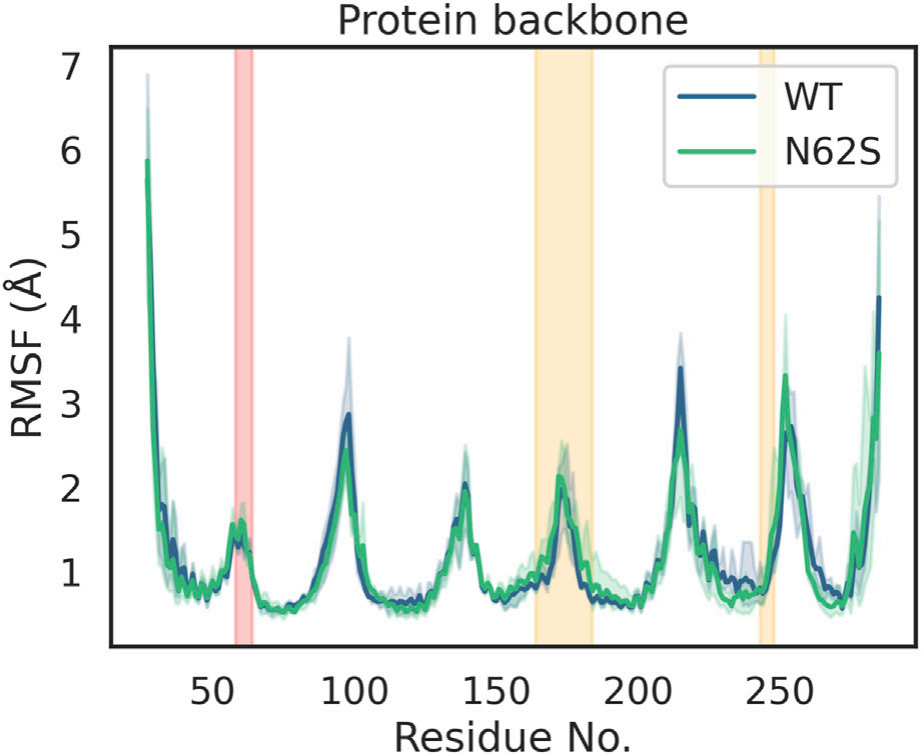
RMSF plot depicting the mean mobility of each residue during the course of MD simulation. ICL1 region, which includes residue 62 affected by the mutation, is marked in red. The residues involved in the formation of ligand-binding subpocket 1 are marked in orange. ICL1; intracellular loop 1; N62S, variant Asn62Ser of MRGPRX2; RMSD, Root mean square distance; WT, wild type of MRGPRX2.

Average structures of the wild-type protein and the N62S variant were calculated as described above. Their superposition is presented in Figure 1. Root mean square deviation (RMSD) plots were generated to show how the protein backbone, as well as some selected residues, change position with respect to the starting point over the course of the simulation. The calculated RMSD values are depicted on Figure 2 as a mean of three independent MD runs. Although differences can be observed in the dynamics of N62S backbone when compared to wild-type, the average RMSD is comparable, with the mean of 2.25 Å for WT and 2.38 Å for variant protein. After the initial ∼200 ns stage of dynamic changes, both of the systems reached equilibrium, which can be identified as the flattening of RMSD curve to more constant level (Figure 2a). Similar to the previous analysis, RMSD plot for α-carbon atoms of subpocket 1 residues revealed that both systems reach equilibrium in ∼200 ns, and no major differences in RMSD values can be observed further on (Figure 2b). The difference in mean RMSD for wild-type and N62S subpocket 1 residues is insignificant and equals 0.26 Å N62 is located in ICL1 (intracellular loop 1) fragment of the protein chain, which connects two (H1 and H2) of the seven helices forming the transmembrane domain of MRGPRX2. Inspection of RMSD plots for ICL1 residues (Figure 2c) reveals no difference in conformation of the mutant loop when compared to wild type. Root mean square fluctuation (RMSF) plot comparing wild-type and N62S trajectories was also generated and is shown in Figure 3. The helix-bundle structure of MRGPRX2 chain A can be directly observed as seven low-mobility regions on the RMSF plot, corresponding to seven transmembrane α-helices connected by highly fluctuating loops. RMSF plot of the mutant protein aligns completely with the wild type, suggesting that the introduction of N62S does not affect the dynamic behaviour of MRGPRX2 chain A helices in a way that may disrupt or alter its function.

## 4 Discussion

In this study, we investigated gene sequences in the MRGPRX2 protein coding region for both patients who experienced immediate hypersensitivity reactions to NMBAs and controls. Analysis revealed the presence of a single missense SNP: N62S located in the first intracellular domain of MRGPRX2, the frequency of which did not differ between the two groups. The frequency of occurrence of N62S in the patient group was independent of the given causal drug.

A separate analysis of a subset of patients without features of an IgE-dependent reaction (negative BAT and sIgE), and therefore most suspect for the possibility of an alternative pathway of mast cell activation (Sabato et al., 2023), also showed no effect of the presence of the detected SNP on the occurrence of the reaction. Mast cell degranulation induced by MRGPRX2 activation has been shown to follow a distinct pattern to that mediated by IgE (Gaudenzio et al., 2016). Therefore, it has been suggested that MRGPRX2-dependent reactions have a milder course with a predominance of cutaneous symptoms (McNeil, 2021). For this reason, we compared patients with mild/moderate POH to patients with severe POH and patients with cutaneous symptoms only to other patients. Also in these subgroups there was no effect of N62S on the clinical course of the reaction.

Furthermore, based on RMSD analysis of the molecular dynamics trajectories obtained for WT and N62S variants, we were able to show that the SNP we describe does not significantly affect the conformation of the MRGPRX2 transmembrane domain as a whole, nor does it affect its binding site. This is consistent with the results of RMSF analysis, which quantifies the degree of mobility (fluctuations) for each of the protein residues, as the RMSF plots for WT and N62S variants overlap. The fact that no major changes can be observed in mean RMSD and RMSF of ICL1 region, where N62 is located, between wild-type and mutant variant can be a proof of protein stability and indicate that no structural changes occur in the chains adjacent to the N62S site.

As mentioned above, some previously identified SNPs in MRGPRX2 gene have been shown to affect the function of this receptor, e.g., loss of activation after stimulation with substance P (Reddy et al., 2017) or enhanced response to ciprofloxacin (Hamamura-Yasuno et al., 2022). Regarding the NMBAs, Suzuki et al. described three mutations (M196I, L226P and L237P) in MRGPRX2 in single patient after POH induced by rocuronium (Suzuki et al., 2020). But, another group showed that these MRGPRX2 variants do not exhibit gain-of-function phenotype for rocuronium-induced MC degranulation (Chompunud Na Ayudhya et al., 2021). Chung et al. detected N62S in MRGPRX2 gene along with seven other SNPs in Korean patients with anaphylaxis induced by iodinated contrast media (Chung et al., 2020). However, similar to our results, the frequency of N62S was not different from the control group.

Most recently, Qi et al. performed a whole-exome sequencing study in a group of Han Chinese patients with a history of perioperative anaphylaxis, including 24 patients with an NMBA-induced reactions (Qi et al., 2023). Three non-synonymous variants of the MRGPRX2 gene were identified, including the N62S. Compared to the control group, these variants did not play a significant role in the induction of perioperative anaphylaxis, which is consistent with our results. The authors included variants with a frequency of ≥0.01 in their analysis. As the estimated frequency of perioperative anaphylaxis ranges widely from 0.003 to 0.00005 (Garvey et al., 2019a; 2019b), less common MRGPRX2 variants may not have been analysed. In our study, we did not apply this limitation and sequencing was performed using the more precise Sanger method. Also given the genetic differences between different ethnic groups, results should not be automatically extrapolated, for example, between Caucasian and Han Chinese groups.

The clinical significance of N62S SNP has been described in patients with ulcerative colitis (UC) (Chen et al., 2021). Serine residue in intracellular loop of MRGPRX2 results in the gain of a phosphorylation site, thereby enhancing β-arrestin recruitment and receptor desensitisation in response to PAMP-12 (proteolytic cleavage product of adrenomedullin), a peptide abundant present in UC. Consequently, N62S cause decreased MCs degranulation due to increased desensitisation of the receptor and for this reason it may be protective in UC (Chen et al., 2021). These results corroborate the later findings of Yang et al. (2022) who generated an MRGPRX2 N62S construct and used it in HEK cells to screen for responses to nearly 3,500 drugs. In validating the assay, they found that the N62S variant in MRGPRX2 resulted in inhibition of intracellular Ca2+ influx upon stimulation with substance P, but did not show that the presence of the variant tested affect the response to any of the NMBAs. A recent clinical study comparing of a control group and a small group of patients who experienced hypersensitivity reactions after taking fluoroquinolones showed that the N62S variant occurs at frequencies of 47.5% and 40%, respectively. These frequencies are highly consistent with our results (Quan et al., 2025). The authors also demonstrated that HEK cells transfected with the N62S variant responded with greater increases in inositol phosphate 1 production and calcium influx when stimulated with antibiotics (vancomycin and ciprofloxacin) compared with WT cells. Further insight into the pathomechanism of the phenomenon under investigation could be gained through similar studies with NMBAs ligands. It should be noted that the final assessment of the pathogenicity of a given gene variant is complex and may also be based on expressivity, phenotypic penetration and familial segregation of the variant (Richards et al., 2015). Furthermore, the intrinsic mechanisms regulating MRGPRX2 activation may differ between human mast cells and experimental laboratory cell lines (Li et al., 2025).

In this study, we obtained results that falsify the working hypothesis regarding the presence of gain-of-function variants that are responsible for NMBA-induced POH in MRGPRX2. Nevertheless, these results address important gaps in an area of our current knowledge that has not been explored so far. They also reduce the risk that analogous studies will be repeated unnecessarily by other teams and, by ruling out one hypothesis, our results directs them towards other research avenues to explain the phenomenon under study. Avoiding the publication of some results creates a systematic bias that distorts the real and complete picture of the research subject, and last but not least the ethical obligation towards the patient participants of the study to appreciate their commitment and effort by making the data obtained public (Sak, 2023; Wolf, 2017; Sandercock, 2012; Teixeira Silva, 2015). Alternative explanations regarding the relationship between NMBA-induced mast cell activation and MRGPRX2 include, e.g., differences in blood levels or in local tissue expression of MRGPRX2, as well as the simultaneous triggering of different pathways that may activate mast cells during anaesthesia (Porebski et al., 2018). This field may also benefit from further, more extensive genetic studies.

In summary, our work does not support one of the most intriguing recent hypotheses concerning the unknown mechanisms of the NMBA-induced POH reactions. It turned out that the only MRGPRX2 SNP we found, namely, N62S, is not clinically relevant for the phenomenon under study. This finding is supported by our molecular dynamics results and the aforementioned *in vitro* study by Yang et al. (2022). It is also consistent with the data of Qi et al. (2023), however, in our study we focused on a larger group of patients after NMBA-induced reactions and analysed patients in terms of severity of symptoms and after subgrouping according to the algorithm (Sabato et al., 2023) into subgroups with classic type I immediate allergy and a potential MRGPRX2-dependent reaction. The presence of this SNP did not promote IgE-independent POH, nor did it affect the severity of the reaction.

It is important to be aware of the limitations of this study. Clearly, neither BAT nor drug-specific IgE determinations have full sensitivity as diagnostic tests (Mayorga et al., 2023). Therefore, some patients may actually have false negative outcomes of testing. On the other hand, the increased expression of MRGPRX2 on the surface of basophils in some individuals may affect BAT results independently of IgE-mediated activation. In addition, the commercial morphine-based test we used detects IgE specific to suxamethonium and rocuronium, which was the most common causative drug in the study group, but is inefficient in detecting atracurium-reactive antibodies (Ebo et al., 2023). Regarding the patients selection, it should be taken into account that sensitization to tertiary and quaternary ammonium acquired through exposure to non-NMBA compounds (e.g., pholcodine) is an alternative explanation for NMBA-induced anaphylaxis without previous exposure. Another limitation of the study is that it did not consider drug-specific IgE for rocuronium in patients with reactions from this culprit.

## Data Availability

The datasets generated for this study can be found in the RODBUK Cracow Open Research Data Repository at https://home.rodbuk.pl/en.
